# Giant lung metastasis of NRAS-mutant melanoma in a 24-year-old patient with a history of BRAF-mutant conventional melanoma harboring Spitzoid morphology: a case report

**DOI:** 10.1186/s13000-020-01046-3

**Published:** 2020-10-25

**Authors:** Jiri Vachtenheim, Roman Kodet, Ondrej Fischer, Vitezslav Kolek, Zuzana Strizova, Andrej Ozaniak, Jan Simonek, Alan Stolz, Jiri Pozniak, Jan Kolarik, Monika Svorcova, Jiri Vachtenheim, Robert Lischke

**Affiliations:** 1 Third Department of Surgery, First Faculty of Medicine, Charles University and University Hospital Motol, Prague, Czech Republic; 2 Department of Pathology and Molecular Medicine, Second Faculty of Medicine, Charles University and University Hospital Motol, Prague, Czech Republic; 3 Department of Respiratory Medicine, Palacký University Medical School and Teaching Hospital, Olomouc, Czech Republic; 4 Department of Immunology, Second Faculty of Medicine, Charles University and University Hospital Motol, Prague, Czech Republic; 5 Department of Transcription and Cell Signaling, Institute of Medical Biochemistry and Laboratory Diagnostics, First Faculty of Medicine, Charles University, Prague, Czech Republic

**Keywords:** Spitz nevus, Atypical spitz tumour, Spitzoid melanoma, Conventional melanoma, Metastasis, Molecular analysis, Case report

## Abstract

**Background:**

Spitzoid melanocytic lesions represent a heterogeneous group of proliferations with ambiguous and overlapping terminology. The exact distinction of a Spitz nevus from a Spitzoid melanoma can be very difficult or, in some cases, impossible. Among the Spitzoid lesions, there is a lesion termed an atypical Spitz tumour (AST) that has intermediate histopathologic features between those of a Spitz nevus and a Spitzoid melanoma and thus uncertain malignant potential. There are several rare cases of patients with a Spitzoid melanoma initially misdiagnosed as a Spitz nevus or an AST with fatal consequences. It is, therefore, advised to perform a molecular characterization in cases where uncertain skin lesions are presented, as it may provide extended set of information with a possible impact on the treatment options. Furthermore, preventive measures, such as regular physical and skin examinations, as well as thorough scheduling of individual follow-up visits, are essential in patients with potentially malignant skin nevi.

**Case report:**

We report a case of a young adult female with a history of AST excision with a negative sentinel lymph node biopsy (SLNB) and insufficient follow-up. Four years after the primary dermatological diagnosis, she presented with a giant tumour in the right hemithorax. Radical en bloc resection of the tumour with right pneumonectomy and resection of the pericardium with reconstruction of the pericardium using mesh was performed. A definitive histopathological examination revealed a metastatic melanoma. The association of the previously diagnosed AST and subsequent appearance of melanoma metastases led to a retrospective re-evaluation of the initial lesion. The suspected diagnosis of Spitzoid melanoma, however, was not confirmed. Moreover, the molecular examination revealed a major discordance between the initial lesion and the lung tumour, which most likely excluded the possible association of the lung metastasis with the initial skin lesion. The initial skin lesion was a BRAF-mutant melanoma with Spitzoid features and termed as AST, while the giant lung metastasis was NRAS-mutant melanoma. The subsequent postoperative course was complicated by the appearance of brain metastases that were stereotactically irradiated. Nevertheless, despite complex specialised medical care, the patient’s clinical condition rapidly deteriorated. By this time, no active oncological treatment was possible. The patient was delegated to local hospice for palliative care six months after the surgery and died three weeks later.

**Conclusions:**

Our patient was surgically treated at the age of 20 for AST and died four years later of metastatic NRAS-mutant melanoma most likely of different occult origin. Molecular characterization, as well as the close clinical follow-up should be always precisely performed in patients with uncertain skin lesions, such as AST.

## Background

A Spitz nevus was originally described by Sophie Spitz in 1948 [1]. This uncommon melanocytic proliferation is characterised by spindled and/or epitheloid nevomelanocytes [2]. More than 70 years after its first description, a Spitz nevus is now classified within a number of Spitzoid lesions and represents a heterogeneous group of proliferations with ambiguous and overlapping terminology.

The spectrum of Spitzoid proliferations ranges from a Spitz nevus, with completely benign biological behaviour, to a Spitzoid (Spitz-like) melanoma, which bears overlapping features of a Spitz nevus and conventional melanoma, carrying a malignant nature.

Among the Spitzoid lesions, there is a lesion termed an atypical Spitz tumour (AST) that has intermediate histopathologic features between those of a Spitz nevus and a Spitzoid melanoma and thus uncertain malignant potential [3].

The proper understanding of the potential risk associated with Spitzoid lesions is of particular importance, especially in tumours displaying features similar to those of a malignant melanoma. However, due to histopathological controversies, the clinical management of Spitzoid lesions remains extremely challenging, and to date, standardized histopathological classification criteria are lacking.

The exact distinction of a Spitz nevus from a Spitzoid melanoma can be very difficult or, in some cases, impossible [4]. Barnhill et al. conducted a blind study in which 30 Spitz-type lesions (including ASTs and Spitzoid melanomas) were reviewed by 10 independent pathologists, and they failed to reach a consensus on the diagnosis, discrimination from a melanoma, and prediction of outcome [5]. Thus, Spitzoid neoplasms are among the most difficult lesions to diagnose in dermatopathology, and a misdiagnosis of a melanoma for a Spitz nevus or an AST is one of the most frequent causes of malpractice lawsuits in surgical pathology and dermatopathology [6, 7]. There have been reported several cases of patients with a Spitzoid melanoma initially misdiagnosed as a Spitz nevus or an AST with fatal consequences [8].

Here, we report a case of a young adult female with a history of AST excision with a negative sentinel lymph node biopsy (SLNB) and insufficient follow-up. Four years after the primary diagnosis, she presented with a giant tumour in the right hemithorax. Radical surgery was performed, and a definitive histopathological examination revealed a metastatic melanoma. The association of the previously diagnosed AST and subsequent appearance of melanoma metastases led to a retrospective further re-evaluation of the initial skin lesion with molecular analysis. However, the suspected diagnosis of metastatic Spitzoid melanoma was not conclusively confirmed. The specific mutation of the BRAF gene was detected in the initial skin lesion. Nevertheless, this mutational pattern was not observed in the giant lung tumour. Moreover, the lung metastasis exhibited the NRAS gene mutation, while the initial skin lesion was NRAS negative. This discordance between the initial lesion and the giant lung tumour most likely excluded the possible association of the lung metastasis with the initial skin lesion originally termed as AST.

## Case presentation

A 24-year-old female patient with a two-month history of cough resistant to antibiotic treatment was further examined at the Department of Pneumology at another medical facility after the appearance of haemoptysis. Computed tomography (CT) revealed a giant tumour in the right hemithorax. Based on the presence of spindle cells and immunohistochemical positivity for S-100 and SOX 10 with negativity for MelanA and HMB-45, a suspicion on diagnosis of malignant peripheral nerve sheath tumour was made at another medical facility. The differential diagnoses comprised synovial sarcoma and malignant solitary fibrous tumour.

Her past medical history included excision of AST localised under right breast at another medical facility four years prior. The tissue sample was described as melanocytic affection displaying patterns of intradermal proliferation of large melanocytes with abundant amphophilic cytoplasm, epitheloid and vesicular nuclei, focally containing prominent nucleoli. A certain proportion of cells was arranged in small nests and isolated mitoses, together with the maturation disorders were seen. A dense lymphoid infiltrate was observed in the stroma. The expression of p21 and p16 was demonstrated, as well as positive staining for Melan A and S-100 protein. By fluorescence in situ hybridization (FISH) method, the evaluation of numerical aberrations in the region of CCND1 (11q13), RREB1 (6p25), MYB (6q23) and centromeric CEP 6 genes was performed and stated negative for all of the above-mentioned regions. According to the evaluating department of pathology, the initial lesion was classified as AST. Therefore, reexcision with margin of 1 cm was performed and involvement of the sentinel lymphatic node was ruled out by an SLNB executed at the same facility. However, the patient did not obtain any information regarding the possible overlap of an AST with another malignant skin lesions at that time, and no follow-up was scheduled.

At the Department of Pneumology, distant metastases were excluded by brain CT with intravenous contrast agent and positron emission tomography/computed tomography (PET/CT) of the trunk. Bronchoscopy displayed extramural compression of airways distal to the tracheal carina. Significant elevation of neuron-specific enolase (NSE) and CA 125 was present.

**Fig. 1:**
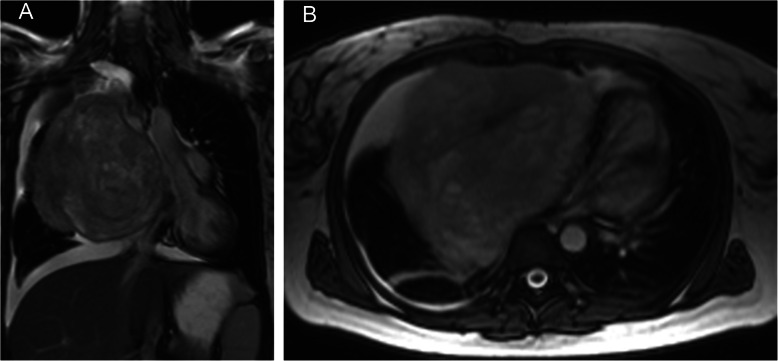
Preoperative cardiovascular magnetic resonance of thorax shows a giant tumour in the right hemithorax. **a** Frontal position with mediastinal shift to the left side. **b** Transverse position

Because of the airway compression and a rapid worsening of clinical symptoms, the patient was immediately transferred to our surgical department with a request for radical surgery. During the examination, the patient exhibited a very low physical performance, dyspnoea at rest, cough, mild haemoptysis with secondary anaemia, borderline respiratory sufficiency, fever, sinus tachycardia, lack of appetite and a history of 10 kg weight loss during the past two months. Operability was promptly evaluated by cardiovascular magnetic resonance (CMR), which verified intimate contact of the tumour to the right atrium and superior vena cava and excluded infiltration of the heart and great vessels (Fig. 1a-b).

**Fig. 2:**
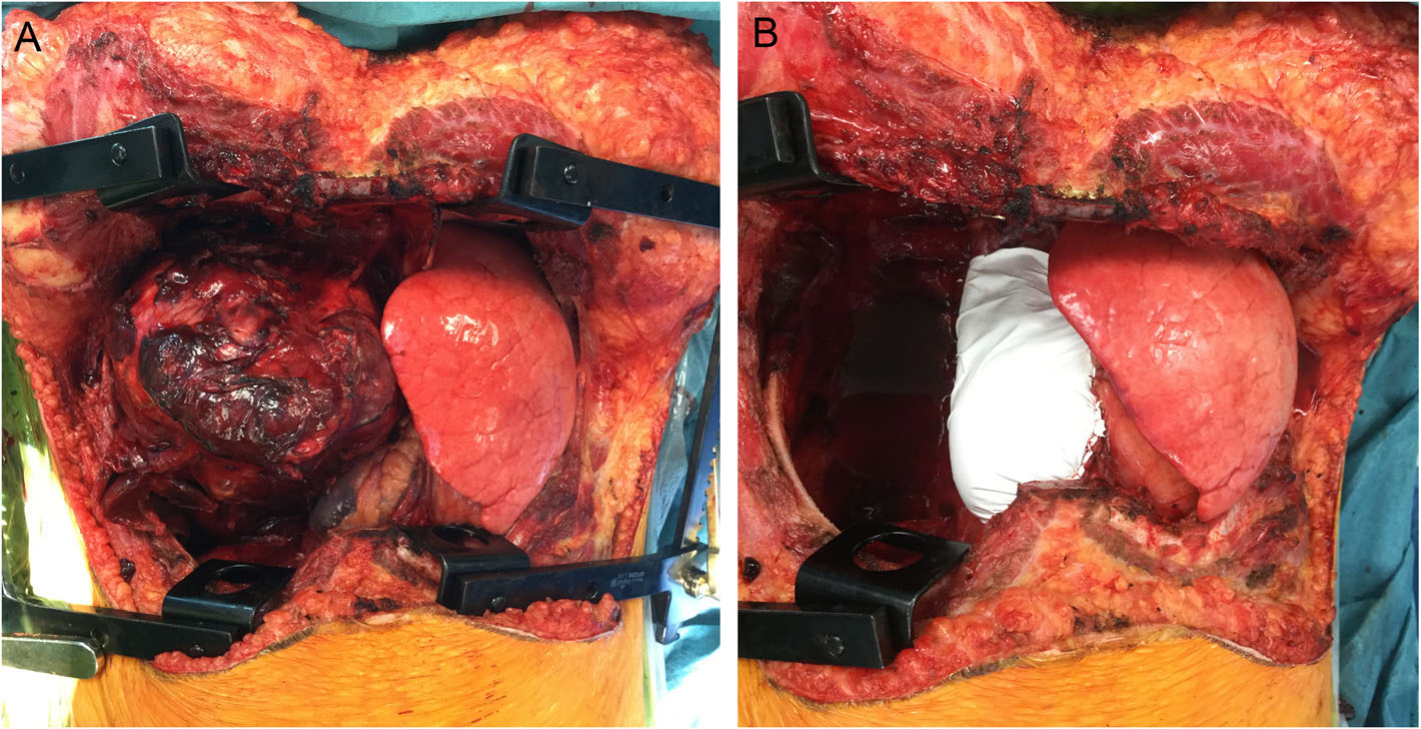
Intraoperative findings – clamshell thoracotomy. **a** Giant tumour of the right hemithorax originating from the right lung. **b** After en bloc resection of the tumour with right pneumonectomy and resection of the pericardium with reconstruction of the pericardium using mesh

Radical en bloc resection of the tumour with right pneumonectomy and resection of the pericardium with reconstruction of the pericardium using mesh was performed (Fig. 2a-b). The postoperative course was uneventful, and the patient was discharged eleven days after the surgery in a very good clinical condition.

Macroscopic examination of the resected specimen showed tumour localised between upper and middle lobe measuring 190 × 120 × 110 mm. The lesion was surrounded by a thin fibrous capsule and was yellowish in colour, lobulated, with pseudocystic transformation, necrosis and hemorrhage on cut surface.

**Fig. 3:**
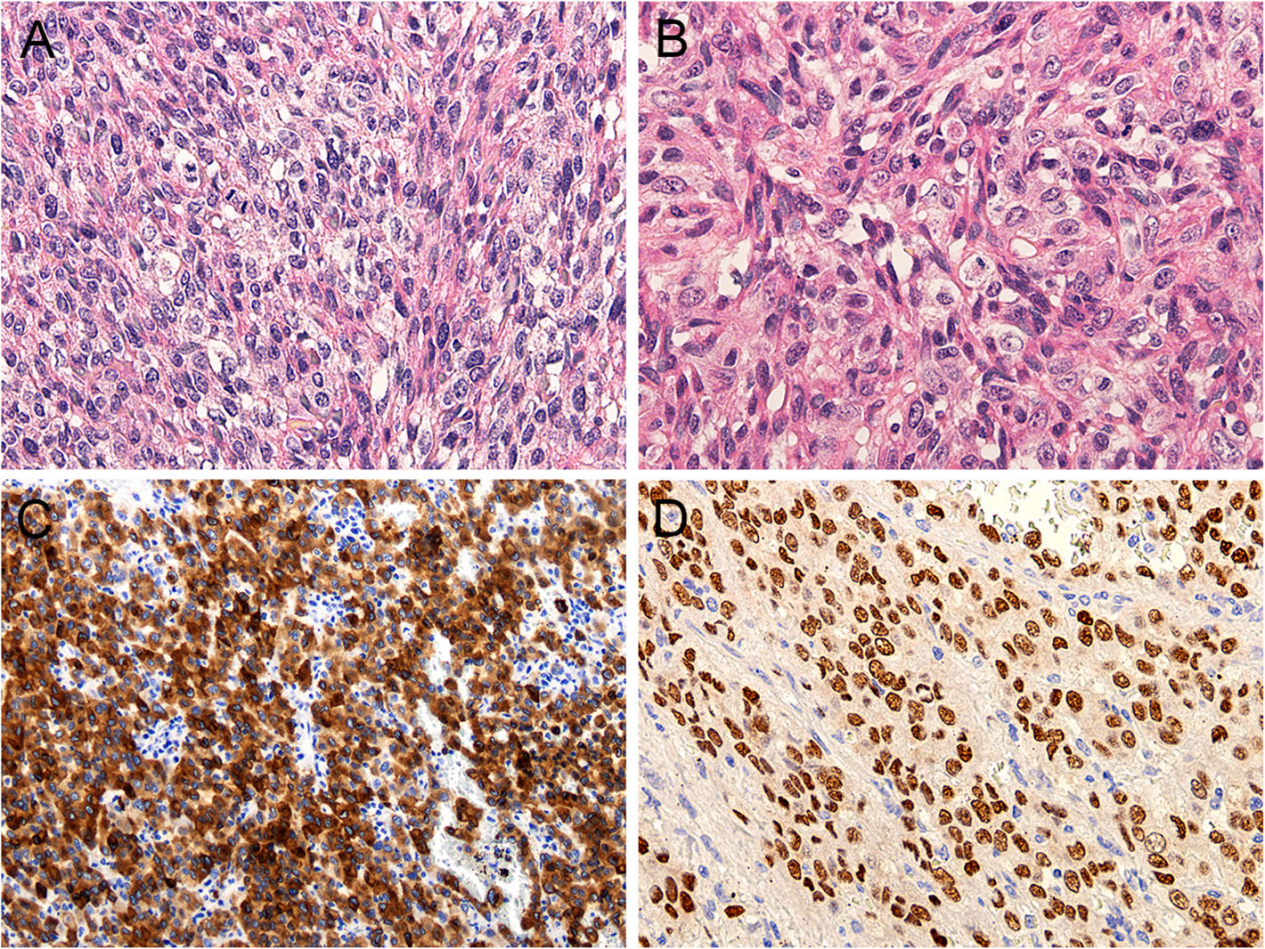
Lung metastatic melanoma – predominantly spindle cell component (**a** H&E stain 200x); epitheloid cell component; no melanin pigment present (**b** H&E staining, 200x). The neoplastic cells are melan A positive, admixt lymphocytes are negative (**c** IHC 100x) and MITF positive (**d** IHC 200x). S100 protein and S0X10 were also strongly positive, whereas HMB45 was negative (not shown)

Definitive histology revealed that the lesion was composed of elongated spindle cells that were arranged in a fascicular or storiform pattern. Also, frequent necroses and haemorrhages were observed. In a minor portion of the lesion, a round cell component was found. The lesional cells had round to oval nuclei of various sizes with clear chromatin, visible nucleoli and a clear to eosinophilic cytoplasm (Fig. 3a-b). Angioinvasion was also present, nevertheless, the visceral pleura and pericardium were not affected.

Immunohistochemistry showed that the tumour cells were positive for Vimentin, CD99, S100, SOX10, MITF, MelanA, INI-1 and Ki-67 (Fig. 3c-d). The tumour cells were negative for desmin, myogenin, myoD1, CK-AE1/3, HMB45, CD57 and GFAP.

Molecular characterization revealed the tumour as negative for the fusion of EWSR1 (22q12) and testing for BRAF exon 15 was negative for the V600E mutation. The DNA isolated from the formalin-fixed/paraffin-embedded (FFPE) sample was analysed by next-generation sequencing (NGS) and an NRAS p.(Q61K) mutation was detected. Mutations in KRAS, HRAS, SMARCA4 and SMARCB1 were not found (Roche NimbleGen Inc., Madison, WI, USA). Fusions involving BRAF, ALK, NTRK1, RET or ROS1 were assessed as negative (ArcherDX, Boulder, CO, USA).

**Fig. 4:**
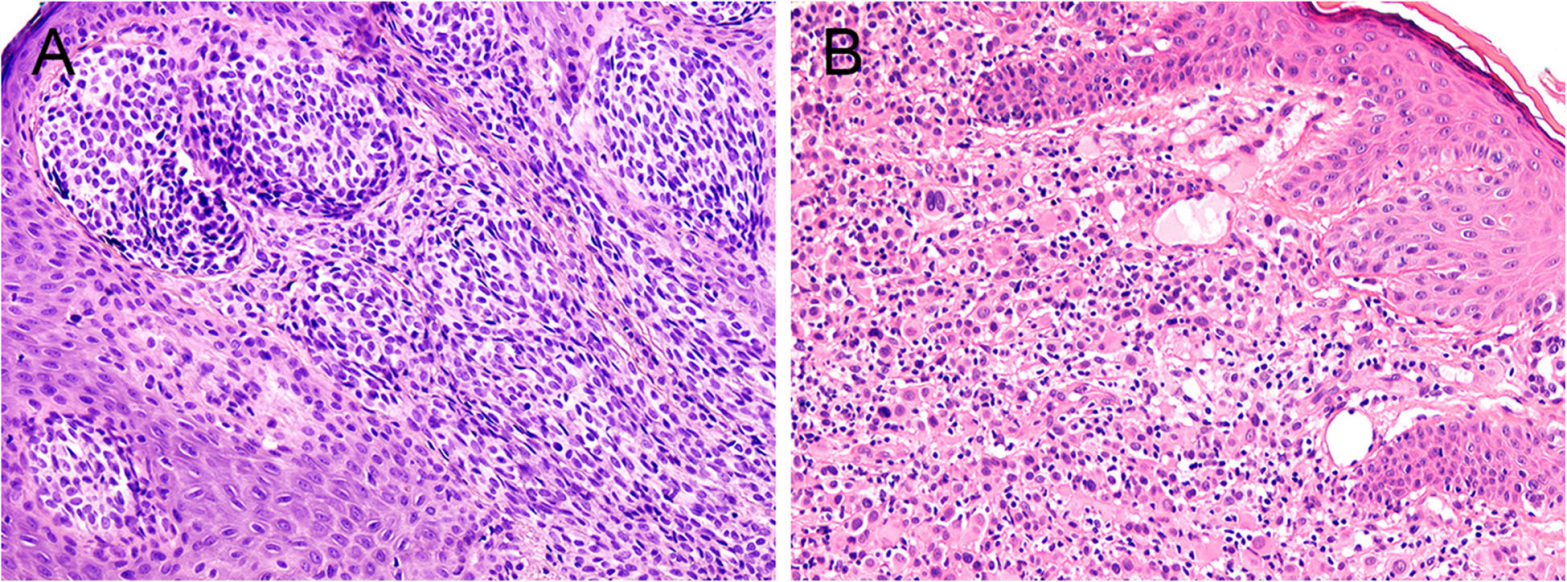
Initial skin lesion, the epidermis uninvolved (**a** H&E stain 100x), epitheloid cell component with atypical cells infiltrating the dermis and partly the epidermis in a different part of the investigated specimen, admixt there are numerous lymphocytes (**b** H&E stain 100x)

Based on the morphological, immunohistochemical (mainly MelanA and MITF positivity) and molecular features, the diagnosis of metastatic NRAS-mutant melanoma was established. Based on the medical history of a previously excised AST, an original biopsy of the primary cutaneous lesion was requested and reviewed at our Department of Pathology. The second-look histopathological examination verified spindle and epitheloid nevomelanocytes arranged in junctional and intradermal nests. Cytologic atypia and numerous mitotic activities were present (Fig. 4 a-b). The lesion was pervaded with lymphoid infiltrate, and pagetoid spread was not demonstrated. The Breslow thickness was approximately 2.7 mm. Immunohistochemistry showed positivity for HMB45, p16, p21, and S100, and the MIB1 proliferation index was 20%. Based on the patient’s clinical course and the appearance of melanoma metastasis, the initial cutaneous lesion was thought to require reclassification as a Spitzoid melanoma with subsequent lung metastasis. However, the molecular examination of the initial lesion revealed a BRAF mutation without the mutation of NRAS gene.

After the establishment of a definitive diagnosis of a metastatic NRAS-mutant melanoma of occult primary site, the patient was planned to be referred to the Department of Oncology. However, the further postoperative course became complicated, and right-sided tonsillitis with methicillin-resistant *Staphylococcus aureus* appeared. Moreover, eight weeks after the surgery, a follow-up PET/CT was performed and revealed multiple new metastases located in the right-sided pleura, left suprarenal gland, thoracic and retroperitoneal lymph nodes, sternal bone and eighth rib on the right. PET/CT also highlighted right palatal tonsillitis, which was presumed to be of staphylococcal origin. The suspected brain metastases were confirmed by immediate brain magnetic resonance, which also showed two small lesions with cerebral oedema.

Corticosteroid antioedematous treatment was given immediately, and the brain metastases were stereotactically irradiated. However, the patient´s condition at that moment did not allow immunotherapy or chemotherapy. Furthermore, dysphagia occurred, and a tumour in the area of the right palatal tonsil was identified.

An otorhinolaryngological examination and biopsy proved a malignant melanoma. The patient underwent tonsillectomy; however, the postoperative course was complicated by left-sided hemiparesis, and further brain CT showed multiple new metastases. Palliative irradiation of brain metastases was performed. Nevertheless, despite complex specialised medical care, the patient’s clinical condition rapidly deteriorated. By that time, no active oncological treatment was possible. The patient was delegated to local hospice for palliative care six months after pneumonectomy and died three weeks later.

## Discussion

Spitzoid lesions are uncommon but extremely diagnostically challenging. The most difficult steps in the management of such a heterogeneous group of cutaneous proliferations are the establishment of histopathological features, prediction of the biologic behaviour and decision whether to apply systemic therapy or minimize aggressive treatment.

AST displays similar histopathological features to Spitz nevus, such as enlarged epithelioid and/or spindled melanocytes with abundant opaque or glass cytoplasm, and also to melanoma, such as asymmetry, lack of maturation deep in the dermis, atypical and frequent mitosis in the dermal component, and a brisk lymphocytic infiltrate [9]. Even though most Spitzoid lesions behave in an indolent manner, both ASTs and Spitzoid melanomas can metastasize.

SLNB positivity in patients with ATS (which is reported in approximately 30–50% of cases) does not predict a poor outcome, and thus, the sentinel lymph node status is of questionable diagnostic and prognostic value in these particular neoplasms [10, 11]. Although the SNLB procedure and complete lymphadenectomy do not provide a clinical benefit, they should be considered in individual patients with challenging Spitzoid lesions [12].

Ancillary diagnostic techniques such as comparative genomic hybridization (CGH), FISH or NGS offer new opportunities for the precise diagnosis. Spitz tumours are defined in the 2018 WHO classification of skin tumours by the presence of specific genetic alterations, such as HRAS mutations or kinase fusions (BRAF, MAP3K8, ROS1, ALK, NTRK1, NTRK3, RET, MET and MERTK) [13]. Activating HRAS mutations are found in approximately 20% of Spitzoid neoplasms, while BRAF and NRAS mutations are mostly found in conventional melanomas. However, further studies reported the presence of BRAF mutations in up to 20% and NRAS mutations in up to 5% of Spitzoid neoplasms. ALK, ROS1, NTRK1 and RET gene fusions were found in 10%, 17%, 16% and 5% of the cases respectively [14].

In our case report, a young woman with a giant metastasis of NRAS-mutant melanoma in the right hemithorax was immediately operated, however, died few months later due to the development of brain and other distant metastases. Chemotherapy, nor checkpoint immunotherapy, were not indicated.

The patient’s medical history of previous AST excision caused a discussion about the AST being origin of lung metastasis and should be therefore reclassified as Spitzoid melanoma as rare cases of patients with a melanoma that was initially misdiagnosed for a Spitz nevus or an AST with fatal consequences have already been reported.

The available data have prompted us to request for the initial skin lesion and re-evaluate the tissue sample, originally termed at another medical facility as AST. IHC showed positivity for HMB45 in the AST, while this marker was negative in the lung metastasis. However, it is known that 25% melanoma metastasis are negative for HMB45 [14]. Further molecular characterisation revealed discordance in BRAF and NRAS mutation in initial AST and lung metastasis, as AST was positive for BRAF mutation and negative for NRAS mutation. Interestingly, intra-patient heterogeneity of BRAF and NRAS molecular alterations between paired primary melanoma and metastasis has been described with observed discordance rate 13,3% [15]. Moreover, a distinct subset of AST with BRAF mutation and loss of BAP1 expression has also been reported [16].

Molecular analysis demonstrated that the lung metastasis most likely did not originate from the initial AST and the patient presented with two different melanocytic lesions in a four-year time period: AST of the trunk and lung metastasis from presumably occult cutaneous melanoma of unknown primary site. Considering molecular characterisation of both lesions, it is rather improbable that there was an association between the initial AST and the giant lung metastasis. However, discordance in BRAF and NRAS mutation between primary lesion and metastasis or AST with BRAF mutation is conceivable. Possibility of primary pulmonary melanoma with no association with the initial AST is highly controversial [17]. However, primary pulmonary melanoma with NRAS mutation has been recently reported [18].

Finally, despite the previously mentioned controversies, presence of BRAF mutation in the skin lesion previously diagnosed at another medical facility most likely rules out a Spitzoid melanocytic neoplasms and assigns the atypical lesion to the group of conventional melanocytic neoplasms. Thus, the most correct diagnosis of the initial skin lesion would be a “conventional” melanoma harboring a Spitzoid morphology [14].

## Conclusion

Our patient was surgically treated at the age of 20 for AST and died four years later of metastatic NRAS-mutant melanoma most likely of different occult origin. However, we aim to highlight that due to the histopathologic controversies regarding Spitzoid lesions, a previous clinical presence of an AST should be a reason for a systematic follow-up. Moreover, precise molecular characterization should be always performed in patients with uncertain skin lesions, such as AST. However, there is not clearly defined distinct set of biomarkers currently used that can with high sensitivity and specificity discriminate Spitz lesions from melanoma or distinguish whether an ambiguous Spitzoid lesion will behave benign or malignant in the future [14].

## Data Availability

All data generated or analyzed in the current article are available from the corresponding author on reasonable request.

## Abbreviations

AST: Atypical Spitz tumour
SLNB: Sentinel lymph node biopsy
CT: Computed tomography
FISH: Fluorescence in situ hybridization
PET/CT: Positron emission tomography/computed tomography
NSE: Neuron-specific enolase
CMR: Cardiovascular magnetic resonance
FFPE: Formalin-fixed/paraffin-embedded
NGS: Next-generation sequencing
CGH: Comparative genomic hybridization
H&E: Hematoxylin–eosin
IHC: Immunohistochemical staining

## References

[1] Spitz S (1948). Melanomas of childhood. Am J Pathol.

[2] Luo S, Sepehr A, Tsao H (2011). Spitz nevi and other Spitzoid lesions: Part I. Background and Diagnoses. J Am Acad Dermatol.

[3] Moscarella E, Lallas A, Kyrgidis A (2015). Clinical and dermoscopic features of atypical Spitz tumors: a multicenter, retrospective, case-control study. J Am Acad Dermatol.

[4] Kim JY, Choi JE, Ahn HH (2012). A case of spitzoid melanoma with lymph node metastasis in a child. J Korean Med Sci.

[5] Barnhill RL, Argenyi ZB, From L (1999). Atypical Spitz nevi/tumors: lack of consensus for diagnosis, discrimination from melanoma, and prediction of outcome. Hum Pathol.

[6] Cho-Vega JH (2016). A diagnostic algorithm for atypical spitzoid tumors: guidelines for immunohistochemical and molecular assessment. Mod Pathol.

[7] Van Dijk MCF, Bernsen MR, Ruiter DJ (2005). Analysis of mutations in B-RAF, N-RAS, and H-RAS genes in the differential diagnosis of Spitz nevus and spitzoid melanoma. The American journal of surgical pathology.

[8] Lee DA, Cohen JA, Twaddell WS (2006). Are all melanomas the same? Spitzoid melanoma is a distinct subtype of melanoma. Cancer.

[9] Cho-Vega JH (2006). A diagnostic algorithm for atypical spitzoid tumors: guidelines for immunohistochemical and molecular assessment. Mod Pathol.

[10] Ludgate MW, Fullen DR, Lee J (2009). The atypical Spitz tumor of uncertain biologic potential: a series of 67 patients from a single institution. Cancer.

[11] Massi D, De Giorgi V, Mandalà M (2016). The complex management of atypical Spitz tumours. Pathology.

[12] Duncan LM (2014). Atypical Spitz tumours and sentinel lymph nodes. Lancet Oncol.

[13] Raghavan SS, Peternel S, Mully TW, et al. Spitz melanoma is a distinct subset of spitzoid melanoma. Modern Pathology. 2020;33:1122–34. 10.1038/s41379-019-0445-z PMC7286778 31900433

[14] Hillen LM, Van den Oord J, Geybels MS (2018). Genomic landscape of Spitzoid neoplasms impacting patient management. Frontiers in medicine.

[15] Pellegrini C, Cardelli L, Padova MD (2020). Intra-patient Heterogeneity of BRAF and NRAS Molecular Alterations in Primary Melanoma and Metastases. Acta Dermato-Venereologica.

[16] Wiesner T, Murali R, Fried I (2012). A distinct subset of atypical Spitz tumors is characterized by BRAF mutation and loss of BAP1 expression. The American journal of surgical pathology.

[17] Yang C, Sanchez-Vega F, Chang JC, et al. Lung-only melanoma: UV mutational signature supports origin from occult cutaneous primaries and argues against the concept of primary pulmonary melanoma. Modern Pathology. 2020;33:2244–55. 10.1038/s41379-020-0594-0 PMC8386291 32581366

[18] Hibiya T, Tanaka M, Matsumura M (2020). An NRAS mutation in primary malignant melanoma of the lung: a case report. Diagnostic Pathology.

